# Hypothalamic Proopiomelanocortin Is Necessary for Normal Glucose Homeostasis in Female Mice

**DOI:** 10.3389/fendo.2018.00554

**Published:** 2018-09-19

**Authors:** Ramiro Alsina, Milagros Trotta, Viviana Florencia Bumaschny

**Affiliations:** ^1^Universidad de Buenos Aires, CONICET, Instituto de Fisiología y Biofísica “Bernardo Houssay” (IFIBIO), Grupo de Neurociencia de Sistemas, Buenos Aires, Argentina; ^2^Universidad de Buenos Aires, Facultad de Medicina, Departamento de Ciencias Fisiológicas, Buenos Aires, Argentina

**Keywords:** proopiomelanocortin, glucose tolerance, insulin sensitivity, diabetes, hypothalamus, Esr1

## Abstract

The arcuate nucleus of the hypothalamus is a key regulator of energy balance and glucose homeostasis. In particular, arcuate proopiomelanocortin (POMC) neurons inhibit food intake, stimulate energy expenditure and increase glucose tolerance. The interruption of insulin or glucose signaling in POMC neurons leads to glucose intolerance without changing energy homeostasis. Although it was previously shown that POMC *neurons* are necessary for normal glucose homeostasis, the participation of POMC *neuropeptide*, by mechanisms independent of energy balance, remains to be demonstrated. To study the role of POMC in the regulation of glucose homeostasis, we performed glucose and insulin tolerance tests in non-obese mice lacking hypothalamic POMC expression. We found that POMC deficiency leads to glucose intolerance and insulin resistance in female mice before the onset of obesity or hyperphagia. Conversely, POMC deficiency does not impair glucose homeostasis in non-obese male mice. Interestingly, females completely normalize both glucose and insulin tolerance after genetic POMC restoration. Next, to further study sex dimorphism of POMC neurons regarding glucose homeostasis, we measured glucose-elicited changes in C-FOS by performing immunofluorescence in brain slices of POMC-EGFP mice. Remarkably, we found that glucose-induced C-FOS expression in POMC neurons is more than 3-fold higher in female than in male mice. Altogether, our results reveal a key role of arcuate POMC in the regulation of glucose homeostasis in females. Since POMC reactivation completely reverses the diabetogenic phenotype, arcuate POMC could be a potential target for diabetes therapy.

## Introduction

Diabetes mellitus is a condition affecting 422 million people all over the world (1). Type 2 diabetes is the predominant type, and it mainly results from excess body weight and physical inactivity (1). In obese patients and mice, type 2 diabetes greatly improves or even reverts after mild body weight loss (2–4). It is accepted that the improvement in glucose homeostasis is a consequence of fat mass reduction, especially in the liver. However, since the hypothalamus is a key regulator of both glucose and energy homeostasis, hypothalamic mechanisms may also be implicated in the anti-diabetic consequences of losing weight (5, 6).

Within the arcuate nucleus of the hypothalamus, proopiomelanocortin (POMC) neurons sense the energy status of the organism integrating peripheral signals such as leptin, glucose and insulin, among others (7). In turn, POMC neurons coordinate responses to maintain energy balance and glucose homeostasis. Since melanocyte-stimulating hormones (α- and β-MSH) derived from POMC peptide are anorexigenic, POMC deficient patients and mice are extremely obese (8, 9).

Regarding the role of POMC neurons in glucose homeostasis, some studies using electrophysiology on brain slices showed that POMC neuron activity can be modulated by changes in extracellular glucose levels (10–12). Moreover, deleting components of the insulin, leptin or glucose signaling cascades in POMC neurons impairs glucose tolerance and insulin sensitivity in genetically engineered mice (10, 12, 13). In addition, glucose induces both *Pomc* mRNA expression as well as α-MSH release from hypothalamic neurons (12, 14). Although the role of POMC *neurons* in glucose homeostasis has been well established, it remains to be elucidated if hypothalamic POMC *peptide* is directly involved, especially considering that these neurons may also co-secrete glutamate, gamma-aminobutyric acid and Cocaine and Amphetamine Regulated Transcript (15).

We have previously shown that hypothalamic POMC deficiency leads both to obesity and type 2 diabetes (2). Interestingly, restoration of POMC expression in extremely obese POMC-deficient mice induces partial body weight loss but complete normalization of glycemia (2). These results suggest that the reestablishment of glucose homeostasis may not only be a consequence of losing weight but also of *Pomc* restoration itself, which would imply a direct protective role of POMC against type 2 diabetes. In the present work, in order to test the role of POMC in glucose homeostasis independently of its role in body weight maintenance, we determine glucose tolerance and insulin sensitivity in non-obese POMC-deficient mice.

## Materials and methods

### Animal care

Mice were kept under standard laboratory conditions, with controlled photoperiod (lights on from 7 a.m. to 7 p.m.), tap water and standard lab chow available *ad libitum*. Mice were weaned at P21. All procedures were approved by the Institutional Animal Care and Use Committee of the School of Medicine, University of Buenos Aires.

### Mouse lines

**Figure 1:**
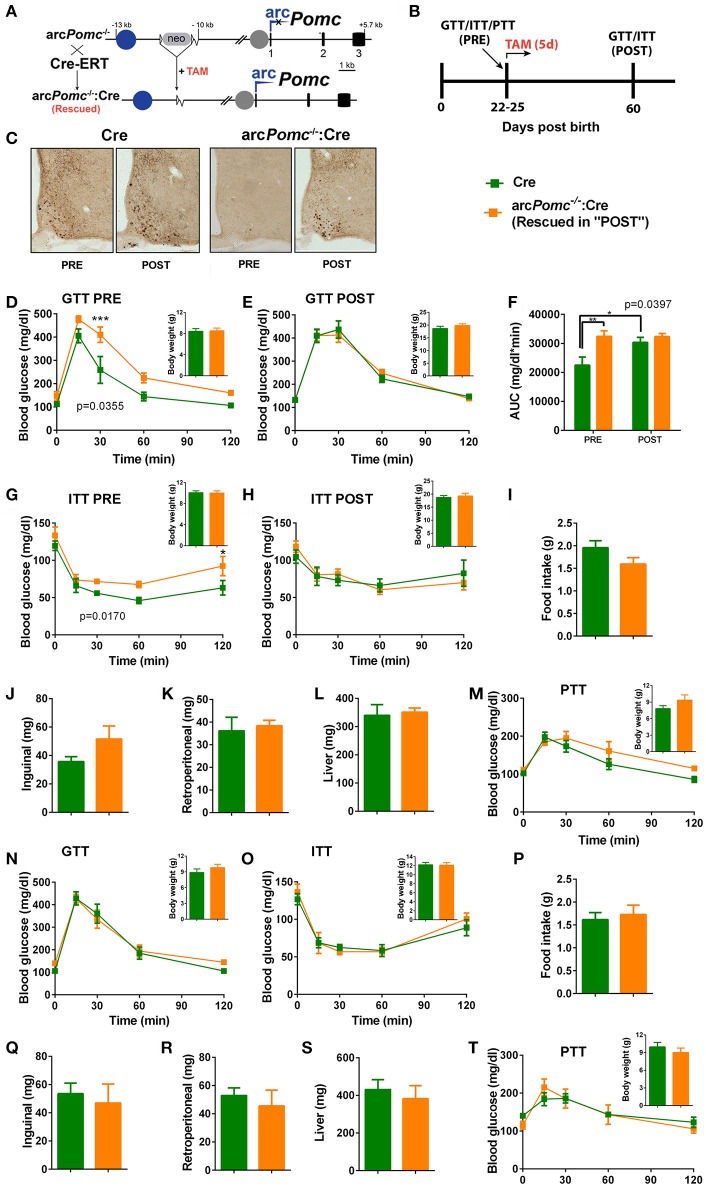
*Pomc* regulation of glucose homeostasis in a mouse model of reversible hypothalamic *Pomc* deficiency. **(A)** A reversible arcuate *Pomc* deficient mouse line (arc*Pomc*^−/−^) was intercrossed with Cre-ERT line to restore *Pomc* expression. arc*Pomc*^−/−^contains an insertion of a neomycin resistance cassette (neo), flanked by *loxP* sites (triangles), interrupting *Pomc* neuronal enhancer activity (Blue circle: nPE1 enhancer; gap after neo: Deleted nPE2 enhancer). Gray circle: intact *Pomc* pituitary promoter. arc: arcuate *Pomc* transcription. Black rectangles: *Pomc* exons. *Pomc* expression can be restored by treating arc*Pomc*^−/−^:Cre mice with tamoxifen (TAM). **(B)** General experimental design. Mice are weaned at P21 and subjected either to Glucose, Insulin or Pyruvate Tolerance Tests (GTT, ITT, or PTT) at P22-P25 (PRE). Mice are treated with TAM for five consecutive days immediately after the first GTT or ITT. At P60, GTT or ITT was repeated (POST). **(C)** Examples of brain coronal sections subjected to immunohistochemistry for POMC in non-treated mice (PRE) and mice treated with TAM (POST). Note that arc*Pomc*^−/−^:Cre mice only show POMC immune-positive neurons after POMC restoration by TAM treatment. Sections correspond to P60-P80 mice. **(D-E)** GTTs and body weights (insets) of females before (PRE) or after (POST) POMC restoration. p: genotype effect of RMA. ^**^p < 0.01 (Bonferroni). *n* = 7–8. **(F)** Area under the curves (AUC) of (**D**,**E**). p: genotype effect of RMA. ^*^p < 0.05*;* ^**^p < 0.01 (Bonferroni). **(G,H)** ITTs and body weights of females before (PRE) or after (POST) POMC restoration. p: genotype effect of RMA. ^*^p < 0.05 (Bonferroni). *n* = 11. **(I)** Daily food intake of female mice measured from P21 to P25. *T*-test, *p* > 0.05. *n* = 8–11. **(J–L)** Weights of retroperitoneal and inguinal fat pads, and livers of female mice at P25. *T*-test, *p* > 0.05. *n* = 6. **(M)** PTTs and body weights of females before POMC restoration. RMA: genotype and genotype x time effects, *p* > 0.05. *n* = 5–6. **(N,O)** GTT and ITT of weanling male mice, respectively. RMA, genotype and genotype x time effect: *p* > 0.05. Insets: body weights. *n* = 6–8 (GTT) and *n* = 7 (ITT). **(P)** Daily food intake of male mice from P21 to P25. *n* = 10–12. *T*-test, *p* > 0.05. **(Q–S)** Weights of retroperitoneal and inguinal fat pads, and livers of male mice at P25. *T*-test, p>0.05. *n* = 5–6. **(T)** PTTs and body weights of males before POMC restoration. RMA: genotype and genotype x time effects, *p* > 0.05. *n* = 5–6. In all graphs, error bars correspond to ±SEM.

**Figure 2:**
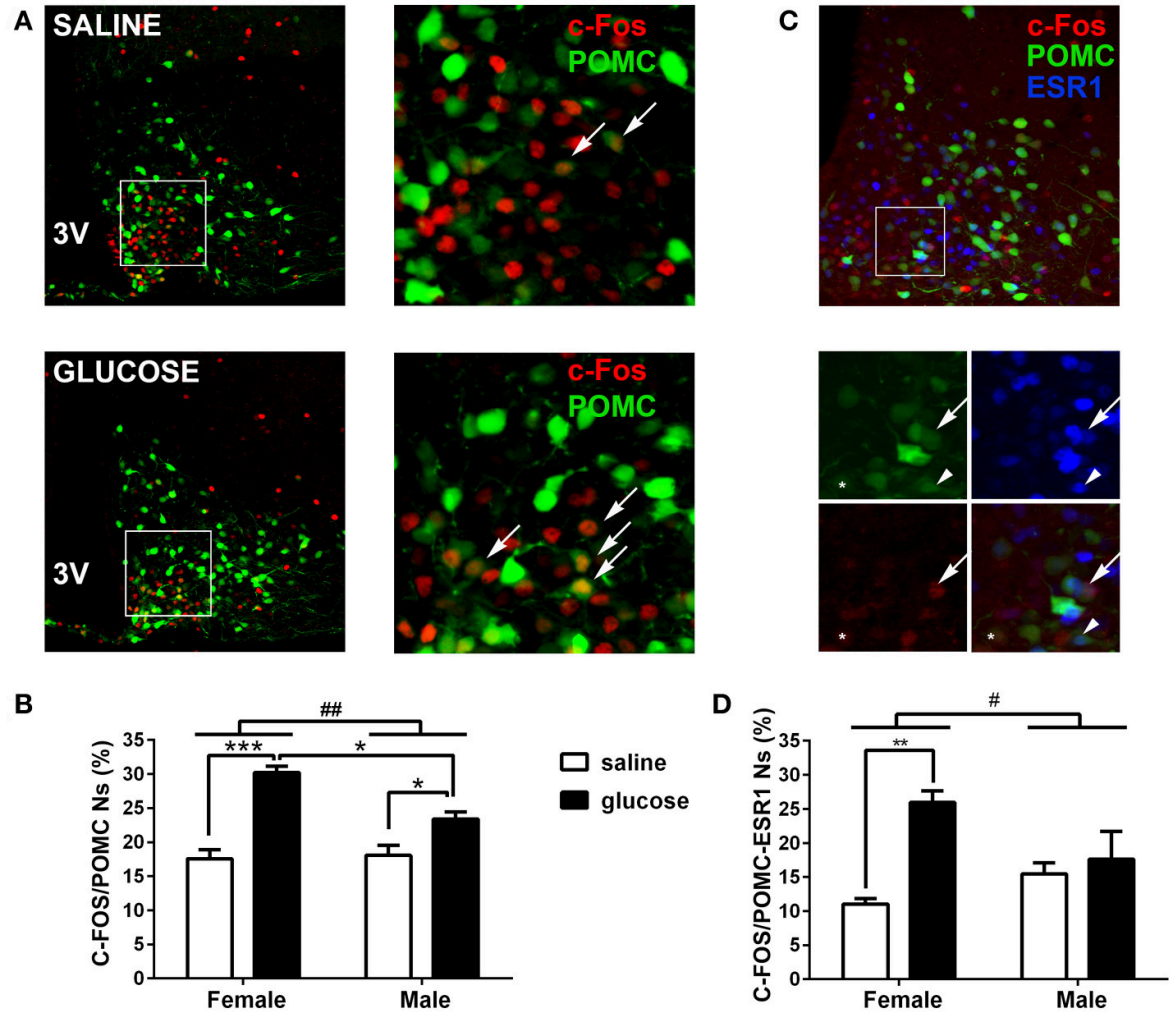
Induction of C-FOS expression by peripheral glucose overload in POMC neurons. **(A)** Representative coronal brain sections of POMC-EGFP female mice injected at P22-25 with saline (top) or glucose (bottom), subjected to anti-C-FOS immunofluorescence. Right panels show magnified images of squared areas depicted in left panels. Green: EGFP expression of POMC neurons. Red: C-FOS immunopositive neurons. Arrows indicate examples of POMC neurons positive for C-FOS. **(B)** Percentage of POMC neurons expressing C-FOS in male and female mice treated either with saline or glucose. POMC neurons per hemi-section: 58.45 ± 10.7; sections per animal: 7 ± 1.7 (mean ± SD). *n* = 4–5. **(C)** Representative coronal brain section of POMC-EGFP female mice injected at P22-25 with glucose (top), subjected to double C-FOS and ERS1 immunofluorescence. Bottom panels show magnified images of squared area depicted in top panel. Green: EGFP expression of POMC neurons. Red: C-FOS immunopositive neurons. Blue: ERS1 immunopositive neurons. Arrows: POMC/C-FOS/ESR1 neuron; ^*^: POMC/C-FOS. Arrowhead: POMC/ESR1 neuron. **(D)** Percentage of POMC/ESR1 neurons expressing C-FOS in male and female mice treated either with saline or glucose. POMC neurons per hemi-section: 50.6 ± 14.1; sections per animal: 6.1 ± 0.9 (mean ± SD). *n* = −3–4. Error bars correspond to ±SEM. ^#^*p* < 0.05 and ^##^*p* < 0.01 (TWA, sex x treatment effect); ^*^p < 0.05, ^**^p < 0.01, and ^***^p < 0.001 (Bonferroni).

arc*Pomc*^−^^/−^ [Figure 1A; (2)], POMC-EGFP (16) and Cre:ERT [B6.Cg-Tg(cre/Esr1)^5Amc^/J] (17) mice were kindly provided by Marcelo Rubinstein, INGEBI-CONICET and bred as previously described (2).

### Glucose, insulin and pyruvate tolerance tests (GTT, ITT and PTT)

Three cohorts of postnatal day 22–25 (P22-25) mice were subjected to GTT, ITT, or PTT (Figure 1B). To avoid excessive weight loss in weanling mice, GTTs and PTTs were performed following 5-h fasting (8 a.m. to 1 p.m.), as suggested (18), and received an i.p injection of glucose (2 g/kg; Sigma) or sodium pyruvate (2 g/kg; Anedra). For ITTs mice were fasted for 2 h (8 a.m. to 10 a.m.) and i.p injected with human insulin (Humulin R; 1 U/kg; Lilly). Blood samples from the tail tip were taken to measure glucose with a One Touch® glucometer (LifeScan, Johnson & Johnson), before and after injections, as described previously (19). Immediately after GTT or ITT, mice were treated with tamoxifen and re-tested at P60.

### CRE induction by tamoxifen administration

Mice were injected i.p. with 50 mg/kg/day tamoxifen (Sigma) during five consecutive days, with a solution of 5 mg tamoxifen/ml of semsame oil (Sigma) prepared as described (20).

### Food intake and fat determination

Another cohort of individually housed arc*Pomc*^−/−^:Cre and Cre mice was used for food intake assessment from P21 to P25. After that, mice were euthanized by cervical dislocation and unilateral subcutaneous (inguinal) and visceral (retroperitoneal) fat pads, as well as livers, were dissected and weighed.

### Immunohistochemistry

Mice were anesthetized with 5% chloral hydrate, perfused with 4% paraformaldehyde and brains were cut into 35 μm coronal sections with a frozen microtome (Leica). Hypothalamic POMC reactivation was confirmed by immunohistochemistry (Figure 1C) using a rabbit polyclonal anti rat-ACTH antibody (1:1,000, A.F. Parlow, National Hormone and Peptide Program, Harbor-UCLA Medical Center) and developed with diaminobenzidine (Vector Labs) as previously described (2). For C-FOS detection, P22-P25 POMC-EGFP mice were fasted from 8 a.m. to 1 p.m., and then i.p injected either with glucose (2 g/kg) or saline. 90-120 min later brains were processed as stated above, immunostained for C-FOS (rabbit anti C-FOS, Merck, 1:1,000) and developed with anti-rabbit-Cy3 antisera (Jackson ImmunoResearch, 1:500). Double immunohistochemistry: (1) mouse monoclonal IgG2a anti C-FOS (Santa Cruz Biotechnologies, 1:4,000) followed by anti-mouse IgG2a-Alexa Fluor 555 antisera (ThermoFisher, 1:1,000); (2) rabbit anti ER alpha antibody (Millipore, 1:10,000) followed by biotinylated anti-rabbit antisera (Vector Labs, 1:200) and developed by streptavidin-Alexa Fluor 647 (Jackson ImmunoResearch, 1:15,000). No staining was performed to visualize POMC neurons since POMC-EGFP mice express EGFP in this neuronal population. Micrographs were taken with an AxioImager M2 motorized fluorescent microscope with Apotome2 structured illumination (Zeiss). Positive neurons were counted using Image J software (21).

### Statistical analysis

All data are presented as the mean ± SEM and were analyzed by Student's unpaired two-tailed *t*-test, one or two way ANOVA (OWA, TWA) or repeated measures ANOVA (RMA), using GraphPad Prism version 6.00 for Windows (GraphPAd Software, La Jolla California, United States). *Post hoc* Bonferroni's test was used when necessary. *P* < 0.05 was considered significant. After RMA, significant Genotype Effect was only taken into account when Interaction (Genotype x Time) was not significant. The total area under the curve (AUC) was calculated using the trapezoidal rule.

## Results

With the aim of studying the role of POMC in glucose homeostasis, we used an arcuate specific *Pomc* knockout mouse model (arc*Pomc*^−/−^) (2). These mice bare a floxed neomycine-resistance gene (*Neo*) immediately downstream of the hypothalamic *Pomc* neuronal enhancer module (Figure 1A). *Neo* cassette prevents arcuate *Pomc* expression while preserving transcription in the nucleus of the solitary tract and the pituitary gland, avoiding corticosterone insufficiency (2).

Arcuate *Pomc* deficiency leads to obesity the fifth week after birth, which predisposes to Type 2 Diabetes in adult mice (2). Thus, in order to dissect the role of POMC in the regulation of glucose homeostasis independently of obesity mechanisms, we performed GTTs in arc*Pomc*^−/−^ mice immediately after weaning, while animals still have normal body weights. Interestingly, we found that despite no significant differences in basal glycemia, female arc*Pomc*^−/−^ are less tolerant to a glucose overload than WT littermates [RMA, genotype effect: *F*_(1, 9)_ = 5.992, *p* = 0.0369, *n* = 5–6 per group].

To further confirm that hypothalamic POMC prevents glucose intolerance, we restored eutopic POMC expression by crossing arc*Pomc*^−/−^ mice with a tamoxifen inducible Cre mouse line (Figure 1A). We have previously shown that POMC recovery at P25 completely prevents hyperphagia and obesity in arc*Pomc*^−/−^:Cre mice (2). Like arc*Pomc*^−/−^, arc*Pomc*^−/−^:Cre female mice showed decreased glucose tolerance despite normal body weight (Figure 1D). However, glucose tolerance is completely normalized in arc*Pomc*^−/−^:Cre after POMC restoration, which further suggests a protective role of POMC against diabetes (Figures 1E,F).

In order to address if glucose tolerance impairment triggered by POMC deficiency is caused by decreased insulin sensitivity, another cohort of arc*Pomc*^−/−^:Cre and Cre female mice were subjected to an ITT. Interestingly, non-obese arc*Pomc*^−/−^:Cre females show decreased insulin sensitivity that was normalized by POMC restoration (Figures 1G,H). Glucose intolerance and insulin resistance of arc*Pomc*^−/−^:Cre females before POMC restoration are not caused by increased inguinal, retroperitoneal or liver fat stores, nor food intake, because no significant differences were found when compared to Cre control mice (Figures 1I–L). Altogether, these results suggest that POMC prevents glucose intolerance by improving insulin sensitivity through mechanisms not related to energy balance regulation or fat storage.

Although it has been previously shown that hypothalamic α-MSH enhances insulin inhibition of hepatic gluconeogenesis in rats (22), we found no significant differences in PTTs between female arc*Pomc*^−/−^:Cre and Cre littermates (Figures 1M).

Contrary to females, non-obese POMC deficient male mice showed normal glucose tolerance and insulin sensitivity, while exhibiting normal food intake, inguinal and retroperitoneal adiposity, liver weights and pyruvate tolerance (Figures 1N–T). To further characterize if POMC neurons also show sexual dimorphism in terms of response to glucose overload, we measured C-FOS immunoreactivity in POMC neurons of POMC-EGFP mice injected either with glucose or saline (Figure 2A). Surprisingly, glucose elicited greater C-FOS expression in arcuate POMC neurons of females than males (Figure 2B). Notably, while C-FOS expression in POMC neurons of glucose treated females was 71.8 ± 5.5% higher than that of saline treated controls, males showed a difference of only 29.5 ± 5.7% (*t*-test: *p* < 0.01). Since a subpopulation of POMC neurons express estrogen receptor alpha (ESR1), which was postulated to control glucose homeostasis (23), we studied C-FOS induction by glucose overload specifically in POMC-ESR1 neurons (Figure 2C). Interestingly, we found that, despite the percentage of POMC neurons expressing ESR1 is similar in males and females (38.9 ± 1.5% and 42.8 ± 1.7%, respectively), glucose significantly increased C-FOS expression only in POMC-ESR1 neurons of female mice (Figure 2D).

## Discussion

In the present work, we studied the regulation of glucose homeostasis by using a reversible POMC knockout mouse model with a unique feature, in which glucose homeostasis and insulin sensitivity can be tested before and after POMC restoration, providing strong evidence for an association between hypothalamic POMC expression and regulation of glucose homeostasis. Our results show that POMC deficiency impairs insulin sensitivity and glucose tolerance in non-obese juvenile female mice. These findings are in line with previous studies showing that the disruption of glucose signaling in POMC *neurons* impairs glucose homeostasis (10, 12, 13). It was also previously shown that POMC deficiency leads to decrease insulin sensitivity in obese and food restricted mice, both with elevated body fat composition (24). In addition, here we found that POMC deficiency leads to insulin resistance and glucose intolerance before the onset of obesity in *ad libitum*-fed female mice characterized by normal food intake, body weight and inguinal, retroperitoneal and liver fat stors. Therefore, the protective role of POMC in glucose homeostasis is presumably achieved by mechanisms independent of those involved in energy balance control. One of the major findings of our study is that POMC recovery completely restores insulin sensitivity and glucose tolerance, which emphasizes the importance of POMC in the regulation of glucose homeostasis. This regulation may be mediated by α-MSH since it was shown that insulin sensitivity is impaired in α-MSH receptor knockout mice through mechanisms involving the sympathetic neural system (25, 26). Regardless the pathway involved, since pyruvate tolerance was unaltered in our model of POMC deficiency, impaired glucose uptake rather than liver glucose production might be responsible for insulin intolerance.

Sexual dimorphism is an interesting feature of POMC neurons concerning the regulation of glucose homeostasis. Here, we found that POMC deletion in hypothalamic neurons leads to glucose intolerance and insulin resistance only in females. Furthermore, glucose-elicited POMC neuron activity is greater in female than in males and the subpopulation of POMC-ESR1 neurons response to glucose only in female mice. However, since glucose was injected systemically, our experiments do not distinguish between a direct or indirect action of glucose in POMC neurons. In either case, we speculate that sexual dimorphism found in our study is a consequence of estradiol (E2) facilitation of POMC activity since it was previously shown that estradiol prevents insulin resistance in POMC neurons of diet induced obese female but not male mice (27). Furthermore, it was demonstrated that E2 increases excitatory inputs, C-FOS protein and *Pomc* mRNA expression in POMC neurons of wild-type females (28, 29). Interestingly, female mice lacking estrogen receptor alpha only in POMC neurons develop insulin resistance and glucose intolerance (23). We hypothesize the existence of a protective pathway linking glucose signaling, ESR1, hypothalamic POMC and glucose homeostasis which is specific to females. Remarkably, women have lower prevalence of diabetes than men despite having higher prevalence of obesity (30). On this regard, our results shed light on the mechanisms underlying gender differences in diabetes pathophysiology.

In summary, we show for the first time a protective role of hypothalamic POMC peptide against glucose intolerance by mechanisms that are independent of POMC role in energy balance. Finally, since *Pomc* reactivation completely reverses the diabetogenic phenotype, arcuate POMC might be a potential target for diabetes therapy, particularly type 2 diabetes.

## Author contributions

VB designed research. RA, MT, and VB performed research. VB contributed new reagents, analytic tools. RA, MT, and VB analyzed data. VB wrote the paper and RA, MT revised it. All authors have approved the final article.

### Conflict of interest statement

The authors declare that the research was conducted in the absence of any commercial or financial relationships that could be construed as a potential conflict of interest.
